# Patient Reported Outcome (PRO) Among High-Grade Glioma Patients Receiving TTFields Treatment: A Two Center Observational Study

**DOI:** 10.3389/fneur.2019.01026

**Published:** 2019-10-01

**Authors:** Julia Onken, Ute Goerling, Marcel Heinrich, Stephanie Pleissner, Dietmar Krex, Peter Vajkoczy, Martin Misch

**Affiliations:** ^1^Department of Neurosurgery, Charité–Universitätsmedizin Berlin, Berlin, Germany; ^2^Berlin School of Integrative Oncology, Charité–Universitätsmedizin Berlin, Berlin, Germany; ^3^Psychooncology, Charité Comprehensive Cancer Center, Berlin, Germany; ^4^Department of Neurosurgery, Universitätsklinikum Carl Gustav Carus, Dresden, Germany

**Keywords:** patient reported outcome (PRO), glioblastoma, TTFields treatment, quality of life (QoL), therapy compliance

## Abstract

**Study design:** A two center, observational study.

**Introduction:** Patient reported outcome (PRO) plays an increasingly important role in the evaluation of novel therapies for tumor patients. It has been shown that tumor treating fields (TTFields) in combination with standard therapy prolong survival in high-grade glioma (hgG) patients. But critics claim that TTFields significantly impacts patients' everyday life due to side effects and average daily time on therapy (18 h) in a patient population with very limited life expectancy and high symptom burden. However, very limited data exist on PRO for TTFields treatment.

**Methods:** This two center, observational study describes PRO of 30 hgG patients receiving TTFields in combination with chemotherapy. We introduced a device-specific questionnaire (DSQ) addressing device-specific restrictions and impact on daily live after 2 months of therapy. Additionally following questionnaires were used: EORTC (European Organization for Research and Treatment of Cancer), QLQ-30 (Quality of life of cancer patients), QLQ BN20 (Quality of life brain cancer module), QLQ FA13 (Cancer-related fatigue), and SSUK-8 (social support).

**Results:** Surveys have been completed by 91% of enrolled patients. EORTC QLQ-30 revealed better physical, emotional, and cognitive function than social and role function of study cohort. TTFields users reported frequently on positive social support and a low level of detrimental interactions. Seventy one percent of patients felt affected in daily life due to TTFields at least 2–3 times per week up to several times per day while maintaining high therapy compliance. Most frequent device-specific restrictions were duration of therapy (74%), size (66%), and weight (70%) of the device and changing time and bonding of the transducer arrays (66%, mean duration: 43.6 min). Restrictions on exercise of hobbies/work (63%/61%), body care (71%), and sexuality/relationship (64%) were most relevant. Seventy percent would recommend TTFields to others and 67% would reuse TTFields treatment again based on their current experience.

**Conclusion:** The study shows that although TTFields treatment frequently affects everyday life in all aspects, therapy compliance was high and 67% of patients would reconsider TTFields for themselves. We propose that findings of PRO be taken into account for medical consultation about TTFields and in future device development to deliver high-value patient-centered care.

## Introduction

Standard treatment of a newly diagnosed high-grade glioma (hgG) involves surgical resection of the tumor followed by concomitant radiochemotherapy and maintenance therapy using the alkylating cytostatic temozolomide (TMZ) (1). With Tumor treating fields (TTFields), an additional treatment modality has been introduced. Its efficacy has been proven in a randomized controlled trial (2, 3). TTFields are alternating electric fields generated by a mobile device system, and applied at low intensity (0.7 V/cm) and medium frequency (200 kHz) via transducer arrays positioned on the shaved scalp in the area of the tumor (TTFields^®^ Instructions For Use, 2016) (4, 5). Highest effectiveness is achieved when using TTFields more than 75% of the time, translating into daily time on therapy of 18 h or more (6, 7). In a recent analysis among participants in the EF-14 study, validated questionnaires from the European Organization for Research and Treatment of Cancer (EORTC) did not show any relevant difference of health related QoL (HrQoL) in patients receiving TMZ plus TTFields or TMZ alone except skin irritations (8). In fact, only 65.8% completed HrQoL assessment at 3 months and only 41.7% of the surviving study participants have been evaluated for HrQoL at 12-months follow up (8). In addition, device-specific restrictions on daily life may not have been translated into the more “general” EORTC questionnaire.

Here, we aim to assess patient-reported outcome (PRO) including motivation for therapy, nature, and frequency of TTFields-specific complaints and their impact on daily life introducing a device-specific questionnaire (DSQ).

## Materials and Methods

### Study Design

Patients were informed about TTFields treatment using the formerly introduced staged approach (9). Between June 2015 and June 2017, patients with hgG including anaplastic astrocytoma and glioblastoma, who have already completed 2 months of TTFields treatment, were included in the PRO-study at two institutions. Thirty out of 33 patients returned the completed questionnaire. None of the patients experienced tumor recurrence/progression during the observation period. Patients received TTFields treatment in addition to first or second line chemotherapy. PRO was routinely assessed with EORTC questionnaires QLQ-30 (Quality of life of cancer patients), QLQ BN20 (Quality of life brain cancer module), QLQ FA13 (Cancer-related fatigue), SSUK-8 (social support), and DSQ 2 months after therapy initiation. Twenty seven patients deemed suitable for TTFields treatment but refused TTFields treatment served as control group. They completed EORTC QLQ-30, QLQ BN20, QLQ FA13, and SSUK-8 2 months after initiation of cyclic TMZ. Local ethic's committee agreed to the study (Vote#EA4/028/17).

### Introduction of a Device-Specific Questionnaire (DSQ)

Since standard EORTC based QoL assessment might not fully reflect TTFields-specific restrictions on daily life, a DSQ has been developed in cooperation with the department of psychooncology, Charité Comprehensive Cancer Center, Berlin, Germany. DSQ has been completed at 2 months of TTFields treatment allowing evaluation of therapy after an adjusting phase. Patients were asked about information resources regarding TTFields treatment and about motivation for therapy. They were queried to retrospectively score subjective changes in QoL before, 1 and 2 months after TTFields treatment at a visual analog scale ranging from 0 to 10 (0 points = poor QoL, 10 points = excellent QoL). We inquired about disturbing factors like nightly operating time, visibility of transducer array, total duration of therapy, financial and administrative procedures, device alarming, side effects (e.g., skin irritation), change and bonding of transducer arrays, battery capacity, size and weight of the device, and head shaving. We specifically requested information regarding when and how often device alarming occurred, what kind of side effects occurred and how often side effects led to therapy interruption. Further, we asked for the time needed for change and bonding of transducer arrays and adaption to therapy. Concerning TTFields impact on everyday life, the following items were assessed: mobility at home and outside, exercises of work, hobbies, housework, shopping, sleep, sexuality/relationship, and motivation for therapy. Individual items could be evaluated with the following answers: very severe restriction (several times per day), severe restriction (several times per week/once a day), moderate restriction (2–3 times per week), mild restrictions (<2–3 times per week), no restrictions or improvement due to TTFields. Furthermore, patients should express their degree of recommendation of TTFields to others and whether they would reconsider TTFields as therapeutic option for themselves at their current knowledge. The English version of the DSQ is attached as Supplementary Material.

### Survey Instruments

The EORTC QLQ-C30 (Version 3.0) provides a comprehensive overview of subscales and multiple items to assess functional integrity, symptom burden, and overall health in oncological patients, based on a total of 30 unique questions on various factors of HrQoL (10, 11). For each subscale and each item, a score is obtained (0–100) which is proportional to the degree of physical and psychological function, to the direct holistic assessment of health status, and to the manifestation of symptoms (10). For assessment of patients' disease-specific quality of life, an add-on module EORTC QLQ-BN20 was used. In order to depict the particular relevance of physical, emotional and cognitive fatigue, EORTC QLQ-FA13 was added (12, 13). The determination of the score and the interpretation of the results for both questionnaires was conducted as described in the literature (10, 14–16). The shortened version of the Illness-specific Social Support Scale SSUK consists of a total of 8 items, of which 4 in the form of a sum score capture the importance of both negative and positive interactions between patients and their caregivers (17, 18).

### Statistical Analysis

Statistical analysis was performed using Microsoft Excel (Microsoft Cooperation, Redmond, WA, USA) and GraphPad Prism 5 (GraphPad Software, La Jolla, CA, USA). The details of the DSQ were predominantly descriptively evaluated. Independent measures of two groups were performed with the Student's *T*-test, independent measures of more than two groups with one-way ANOVA Bonferroni multicomparison test. For non-parametric data, summary data was given as means, median and range. 95% confidence interval (95% CI) were given, significance level was set at *p* < 0.05. Analysis of quality of life was carried out according to the EORTC manual. For global quality of life scale and the functional scales; higher scores correspond with higher functioning and so better quality of life (19). Whereas, for symptom scales and single items; higher scores correspond with more symptoms, high distress and impairment, and indicate a worse quality of life. A difference of more than 10 points was considered clinically meaningful (15).

## Results

### Study Cohort

Thirty patients with diagnosis of a hgG were included in the study (Glioblastoma *n* = 28, anaplastic astrocytoma *n* = 2). Mean age was 50 years (median: 52 years, range: 36–64), male predominance was present with 67%. IDH was mutated in 13% of cases, MGMT promoter methylation was apparent in 43%. Gross total tumor resection was achieved in 80%. Fourteen patients started TTFields in combination to first line chemotherapy (cyclic TMZ). Therapy compliance was on average 83% (range 40–97%) during the first month and 85% (range 56–97%) during the second month of TTFields treatment. The control group consisted of 27 patients with glioblastoma receiving standard treatment with concomitant radiochemotherapy and cyclic TMZ. Detailed patients characteristics of intervention group and control group are given in Table 1.

**Table 1:** Patients' characteristics.

	**TTFields group *n* = 30**	**Control group *n* = 27**
**Age (mean)**	50 years	47 years
**Gender** ***n*** **(%)**		
Female	10 (33%)	8 (30%)
Male	20 (67%)	19 (70%)
**Histology**		
Glioblastoma	28 (93%)	27 (100%)
Anaplastic astrocytoma	2 (7%)	–
**Stage of disease**		
1st line	14 (47%)	27 (100%)
2nd line	16 (53%)	–
**Combined therapy**		
cTMZ	14 (47%)	27 (100%)
mTMZ	6 (20%)	–
CCNU	4 (13%)	–
BEV	1 (3%)	–
PC	1 (3%)	–
NA	4 (13%)	–
**IDH status**		
Wildtype	21 (70%)	2 (7%)
Mutant	4 (13%)	1 (4%)
NA	5 (17%)	24 (89%)
**MGMT status**		
Methylated	13 (43%)	1 (4%)
Unmethylated	12 (40%)	2 (7%)
NA	5 (17%)	24 (89%)
**Extend of resection**		
Gross total resection	24 (80%)	–
Biopsy/partial resect.	5 (17%)	–
NA	1 (3%)	27 (100%)

*BEV, Bevacizumab; CCNU, Lomustine; cTMZ, cyclic Temozolomide; IDH, Isocitrate dehydrogenase; MGMT, O6-methylguanin-DNA-methyltransferase; mTMZ, metronomic Temozolomide; NA, Not Assessed; PC, Procarbazine and Lomustine*.

**Figure 1:**
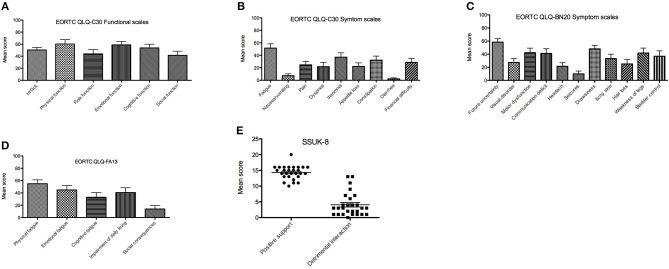
Results of EORTC QLQ-C30, EORTC QLQ-BN20, and EORTC QLQ-FA13 assessed at 2 month of TTFields treatment. Mean scores (X-axis) range from 0 to 100%, which are proportional to the degree of function or symptom load. In the function scales higher scores represent a better level of functioning while in the case of symptom scales/items higher scores mark a higher level of symptomatology or problems. **(A)** Health related quality of life (HRQoL) and functional scales items from EORTC QLQ-C30 2 months after TTFields start. **(B)** EORTC QLQ-C30 symptom scale 2 months after TTFields start. **(C)** EORTC QLQ-BN20 2 months after TTFields start. **(D)** Presence of fatigue assessed with EORTC QLQ-FA13 2 months after TTFields start. **(E)** Social support and detrimental interaction assessed with SSUK-8 2 months after TTFields start. EORTC, European Organization for Research and Treatment of Cancer; QLQ-BN20, Quality of life brain cancer module; QLQ-C30, Quality of life of cancer patients; QLQ-FA13, cancer-related fatigue.

Mean score of HrQoL assessed with EORT QLQ-C30 was 50.3 in the intervention group and 45.7 in the control group (*p* = not significant). The lack of future prospects for glioblastoma patients was one of the central brain tumor-specific factors, which had a highly negative impact on HrQoL in both study groups. Patients who received additional treatment with TTFields showed better emotional function compared to the control group (^**^p < 0.01). At the same time, patients had a significantly lower incidence of insomnia and loss of appetite (*p* < 0.01) and, to a lesser extent, pain, dyspnea, nausea, and vomiting (*p* < 0.05). Results of the EORTC QLQ BN-20 showed no significant differences between groups in means of severity of neurological symptoms due to brain tumor disease. Patients of the intervention group showed clinically relevant better physical, emotional, and cognitive function compared to role function and social function (EORTC QLQ-C30, Figure 1A). The following symptom scale items affected HrQoL the most: fatigue followed by insomnia, constipation, and financial difficulties (EORTC QLQ-C30, Figure 1B). Brain tumor specific concerns/symptoms were in descending frequency: future uncertainty, drowsiness, motor dysfunction/weakness of legs, and communication deficits (EORTC QLQ-BN20, Figure 1C). Cognitive fatigue affected less severely HrQoL than emotional and physical fatigue (EORTC QLQ-FA13, Figure 1D). TTFields users reported frequently on positive social support and a low level of detrimental interactions (SSUK-8, Figure 1E). Although, no significant difference was detected in terms of stressful interactions with the social environment, intervention group received more positive social support in their daily lives relative to the control group.

### Motivation for TTFields and PRO

Eighty one percent of patients were initially informed about TTFields treatment by their treating oncologist/neurosurgeon/radiooncologist. Eleven percentage received information about TTFields via internet or patient platforms. The decision for TTFields treatment was mostly motivated by the feeling of actively doing something against the tumor (100%), the proposed additive therapeutic effect of TTFields to standard therapy (73.3%), the new therapeutic concept (76.6%), and recommendation of the treating physician (96%).

**Figure 2:**
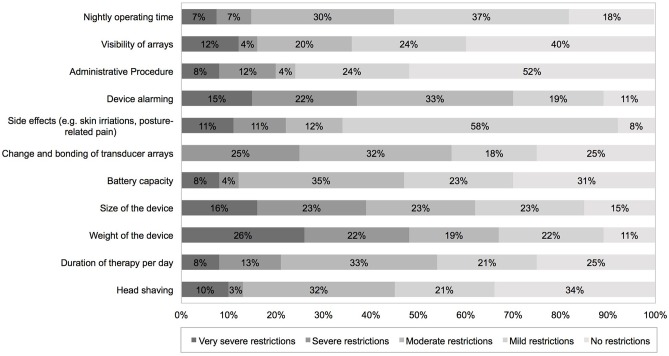
PROMs using a device-specific questionnaire completed 2 months after TTFields initiation addressing device-specific complains. PROMs, patient-reported outcome measures.

Patients needed an average of 24 days (median: 14 days, range: 0–90 days) to adapt to the therapy. Patients retrospectively rated their QoL since the start of TTFields treatment. They reported on a decrease of QoL 1 month after TTFields initiation, which normalized in 90% of cases to initial status after 2 months of TTFields treatment. Most frequent side effects written in the answer portion of the DSQ were skin irritations (40%), such as pruritus, erythema, and secondary efflorescence among the transducer arrays as well as posture-related complications (e.g., back pain due to carrying the device in a single shoulder strap bag, 3%). In addition, 10% felt a causal relationship of psychological complaints such as depression and nervousness under TTFields. As a result of these adverse effects, 6 patients performed an average of 2.7 therapy breaks. Detailed information on usability and side effects are given in Table 2. The following device-specific restrictions had a moderate to very severe impact on everyday life in the majority of our patients: size (62%) and weight (67%) of the device, changing time and bonding of the transducer arrays (57%, mean duration: 47 min, median: 30 min, range 15–150 min) and daily duration of therapy (54%). Device alarming was rated moderate to very severely disturbing in 70%. Alarms occurred on average 3.5 times during the day and 2.0 times during the night. The following topics were rated to not or mildly restricting everyday life in the majority of patients: head shaving (55%), visibility of transducer arrays (64%), side effects like skin irritations (65%), nightly operating time (67%), and therapy costs (76%) (Figure 2).

**Table 2:** Usability and side effects of TTFields.

	**MV**	**Range**
**Frequency of device alarming**		
Day time	3.5	1–10
Night time	2.0	1–20
**Time needed for bonding of arrays (min)**	47	15–150
**Side effects**		
Skin irritations	40%	–
“Electro shocks”	3%	–
Posture-related complications	3%	–
Psychological complaints	10%	–

**Figure 3:**
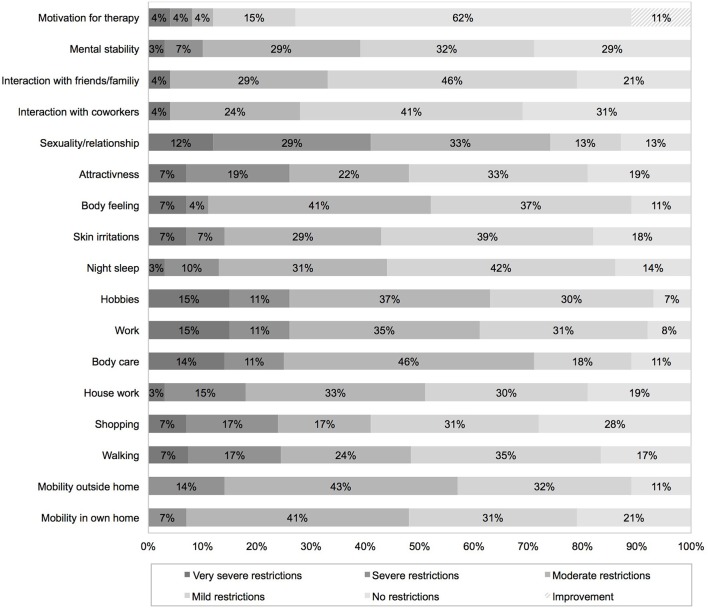
Impact of TTFields on everyday life assessed with a device-specific questionnaire completed 2 months after therapy initiation.

Seventy one percent of all patients faced moderate to very severe restrictions on everyday life due to TTFields treatment. TTFields caused moderate to very severe restriction in exercise of hobbies (63%) and work (61%), and mobility outside home (57%). Own body care was significantly affected in 71%. Seventy four percent reported on moderate to very severe restrictions in sexuality and relationship. Mild or no restrictions were reported on mental stability (61%), interaction with friends/family (67%) and coworkers (72%). Eleven percentage reported increased motivation for therapy due to TTFields treatment. Detailed data is given in Figure 3. Despite the mentioned restrictions, 70% would recommend TTFields to others, 67% would reuse TTFields treatment again based on their own experience. 16.5% would not repeat TTFields treatment, 14.8% did not share their opinion on the topic.

## Discussion

The principal novel findings of the study are that PRO revealed moderate to very severe restrictions on daily living due to TTFields in 71% and a transient subjective decline in QoL during the first month of therapy. Major restrictions existed in exercise of hobbies/work, own body care and sexuality/relationship. Device specific complaints were mostly related to device alarming, change and bonding procedure of arrays, and size and weight of device. However, these restrictions seem to be balanced by positive social support, a positive motivation for actively taking part in therapy and do not translate into poor symptom scales or worsened QoL compared to a time point before starting TTFields.

Patients reported an average time of 24 days to adapt to the therapy, which is reflected in the temporary decline of QoL 1 month after TTFields initiation. After the adaption phase, only 10% reported on a persistent impairment in QoL due to TTFields treatment. Overall, QoL of the study cohort assessed with standardized EORTC questionnaires showed comparable results to other studies (8, 20, 21). In contrast to Taphoorn et al., reduced role and social function had the strongest impact on QoL in our study cohort, which might be a result of the above mentioned restrictions due to TTFields on daily life (8). Medically treatable symptoms such as nausea/vomiting, insomnia, appetite loss, pain, seizures, and headache were relatively well-controlled in our cohort compared to the general population normative data and our control group (22), which could be explained by increased medical and social attention due to TTFields treatment and selection bias. Device support specialists who support the patients at home may have picked up symptoms earlier than at the next physician appointment leading to earlier symptom detection and therapy. Good emotional functioning and high social support within the study cohort might also have contributed to good symptom control and, in turn, confirm proper patient selection (9). Although TTFields treatment frequently affects daily life in all aspects, 67% would reconsider TTFields for themselves and 11% reported improved motivation due to TTFields therapy. However, 1 out of 6 patients would not consent to treatment again leaving room for improvement on the device regarding overall practicability (arrays, batteries, carrying the device). Our study also has some limitations, which restrict the interpretation of our data: (i) usage of a non-validated questionnaire; (ii) missing baseline and long term follow up on QoL; and (iii) non-randomized study with potential selection bias. For this reason, prospective randomized controlled trials that provide the same level of medical and social attention to the control group are important to objectify QoL and therapy response. However, understanding the patient perspective on newly introduced, oncological therapies like TTFields is integral to delivering high-value patient-centered care.

In summary, we propose that PRO of our study be taken into account for medical consultation about TTFields and in the future development of the device. Results of PRO might help to better inform patients about suspected restriction and in turn increase acceptance and compliance toward TTFields treatment.

## Data Availability Statement

The datasets generated for this study are available on request to the corresponding author.

## Ethics Statement

This study was approved and performed in accordance with the University of Berlin, Chartié institutional ethics committee (#EA4/028/17). All subjects gave written informed consent by completing the questionnaires.

## Author Contributions

JO: data acquisition, statistics, and manuscript writing. UG: designs of device specific questionnaire, statistics, and review of the manuscript. MH: data acquisition and statistics. SP: data acquisition. DK: review of the manuscript and data acquisition. PV: review of the manuscript. MM: data acquisition, statistics, manuscript writing and review and study coordinator.

### Conflict of Interest

The authors declare that the research was conducted in the absence of any commercial or financial relationships that could be construed as a potential conflict of interest.
