# Supplementary material for: Patient Reported Outcome (PRO) Among High-Grade Glioma Patients Receiving TTFields Treatment: A Two Center Observational Study
Source: Front Neurol. 2019 Oct 1;10:1026. doi: 10.3389/fneur.2019.01026 (PMC6797850; doi:10.3389/fneur.2019.01026)
Supplement: Supplementary file 1 [file Table_1.DOC]

1 How did you find out about Optune®?

| Physician  (Neurosurgeon, Radiation-Oncologist, Oncologist) | Internet research  (Search-engine) | Online-forum  (e.g. German Brain Tumor Support) | Informative Meeting (e.g. Brain Tumor Day) | Support group | Other patients | Other source  (Please name)? |
| --- | --- | --- | --- | --- | --- | --- |
|  |  |  |  |  |  |  |

2 Which reasons made your decision in favor of Optune® therapy?

|  | Very important | Important | Neutral | Unimportant | No significance at all |
| --- | --- | --- | --- | --- | --- |
| Possibility, to actively do tumor therapy oneself |  |  |  |  |  |
| Effect in addition to standard therapy |  |  |  |  |  |
| New concept, new mechanism of action |  |  |  |  |  |
| Suggested by treating physician |  |  |  |  |  |
| Suggested by other caregivers (friends, family, peers) |  |  |  |  |  |
| Company Information material (brochure, web-page) |  |  |  |  |  |
| „Nothing to lose“ |  |  |  |  |  |
| Few side effects to expect |  |  |  |  |  |
| Others?  Please name |  |  |  |  |  |

**3 Which technical facts made your decision in favor of Optune® therapy?**

|  | Very important | Important | Neutral | Unimportant | No significance at all |
| --- | --- | --- | --- | --- | --- |
| Continuous hair shaving |  |  |  |  |  |
| Therapy duration overall / per day |  |  |  |  |  |
| Weight of the device |  |  |  |  |  |
| Size of the device |  |  |  |  |  |
| Battery capacity of the device |  |  |  |  |  |
| Mains operation / socket - being independent of battery use |  |  |  |  |  |
| Profile of side effects  (e.g. skin irritation) |  |  |  |  |  |
| Total duration of therapy |  |  |  |  |  |
| Suggestion / advice of treating physician |  |  |  |  |  |
| Patient support program of Novocure to cover treatment costs |  |  |  |  |  |
| Availability of detailed information material |  |  |  |  |  |
| Availability of device support specialist |  |  |  |  |  |
| Internet research |  |  |  |  |  |
| Others?  Please name |  |  |  |  |  |
| Possibility, to actively do tumor therapy oneself |  |  |  |  |  |

**4 What aspects of Optune® therapy did you feel to be *disturbing***?

|  | Disturbs very frequently (e.g. often daily/ a few times per day) | Disturbs often (e.g. once daily up to a few times per week) | Disturbs sometimes (e.g. 2-3 times per week) | Disturbs rarely | Disturbs not at all |
| --- | --- | --- | --- | --- | --- |
| Continuous hair shaving |  |  |  |  |  |
| Therapy duration overall / per day |  |  |  |  |  |
| Weight of the device |  |  |  |  |  |
| Size of the device |  |  |  |  |  |
| Battery capacity of the device |  |  |  |  |  |
| Changing arrays |  |  |  |  |  |
| Profile of side effects  (e.g. skin irritation) |  |  |  |  |  |
| Device alarms |  |  |  |  |  |
| Cost coverage by insurance company or Novocure |  |  |  |  |  |
| Total duration of therapy |  |  |  |  |  |
| Array visibility for others |  |  |  |  |  |
| Possibility to use device @ nighttime (noise of device, cable position) |  |  |  |  |  |
| How many alarms did you experience within 24h during daytime? |  | | | | |
| How many alarms did you experience within 24h during nighttime? |  | | | | |
| How much time do you need on average to change arrays? |  | | | | |
| Planned therapy breaks during day or during nighttime? |  | | | | |
| Others?  Please name |  | | | | |

**5 How did factors in section 4 influence daily life?**

|  | No restriction on daily life | Some restriction on daily life | Frequent / intermediate restriction on daily life | Frequent but not severe restriction on daily life | Severe restriction on daily life |
| --- | --- | --- | --- | --- | --- |
| Continuous hair shaving |  |  |  |  |  |
| Therapy duration overall / per day |  |  |  |  |  |
| Weight of the device |  |  |  |  |  |
| Size of the device |  |  |  |  |  |
| Battery capacity of the device |  |  |  |  |  |
| Changing arrays |  |  |  |  |  |
| Profile of side effects  (e.g. skin irritation) |  |  |  |  |  |
| Device alarms |  |  |  |  |  |
| Cost coverage by insurance company or Novocure |  |  |  |  |  |
| Total duration of therapy |  |  |  |  |  |
| Array visibility for others |  |  |  |  |  |
| Possibility to use device @ nighttime (noise of device, cable position) |  |  |  |  |  |

**6 Effect of Optune® therapy on daily life**

**Questions in this section will inquire things that you might do more often or less frequently while using Optune®. Please try to answer these questions independently of symptoms that are caused by the location of the tumor. Please try to only assess things attributed to Optune® use.**

|  | No restriction on daily life | Some restriction on daily life | Frequent / intermediate restriction on daily life | Frequent but not severe restriction on daily life | Severe restriction on daily life | Improvement while using Optune® |
| --- | --- | --- | --- | --- | --- | --- |
| Mobility at home (apartment / house) |  |  |  |  |  |  |
| Mobility away from home (outside house or apartment) |  |  |  |  |  |  |
| Longer walks |  |  |  |  |  |  |
| Walk short distances (e.g. grocery shopping) |  |  |  |  |  |  |
| Housekeeping / Child care |  |  |  |  |  |  |
| Personal hygiene |  |  |  |  |  |  |
| Work / daily chore |  |  |  |  |  |  |
| Hobbies / free time |  |  |  |  |  |  |
| Sleeping @ night |  |  |  |  |  |  |
| Skin symptoms caused by arrays |  |  |  |  |  |  |
| Sense of own body |  |  |  |  |  |  |
| Attractiveness for spouse |  |  |  |  |  |  |
| Relationship / sexual life |  |  |  |  |  |  |
| Daily contact with family / caregivers / relatives |  |  |  |  |  |  |
| Daily contact with friends / colleagues |  |  |  |  |  |  |
| Inner peace / balance |  |  |  |  |  |  |
| Motivation for tumor therapy |  |  |  |  |  |  |
| Pain / itching caused by arrays |  |  |  |  |  |  |

**7 Optune® in everyday life**

**Are there any things that you are able to do with more ease or that require more effort while using Optune®? Are there things / activities you do more often / less often since starting therapy?**

**Please try to answer these questions independently of symptoms that are caused by the location of the tumor.**

**Please name these things.**

**Please rate these things concerning your quality of life?**

**How did you rate your quality of life before starting Optune®?**

**Excellent _____________________________________________________________ Poor**

**How did you rate your quality of life one month after starting Optune®??**

**Excellent _____________________________________________________________ Poor**

**How did you rate your quality of life beyond the 1st month of Optune® therapy?**

**Excellent _____________________________________________________________ Poor**

**Did you need an „adjusting phase“to gain routine in Optune® therapy? If yes, how long did this take?**

**Were there therapy side effects? If yes, please name?**

**Did these side effects cause a break in therapy?**

**If yes, how frequent and how long were these breaks?**

**Would you recommend Optune® therapy to other patients?**

**Yes _____________________________________________________________ No**

**At your current knowledge about Optune® therapy, would you do this therapy again?**

**Yes _____________________________________________________________ No**

**If you experienced disturbances / restrictions while using Optune® therapy, are these „obstacles“ outweighed by possible longer tumor control?**

**Yes _____________________________________________________________ No**

**How old are you?**

**Female / male?**

**Primary therapy / Recurrence therapy?**

**Where was therapy initiated and by whom?**

**We would like to thank you for participating in this survey. The answers to these questions will help to further improve therapy.**
